# The Mouse Grimace Scale: A Clinically Useful Tool?

**DOI:** 10.1371/journal.pone.0136000

**Published:** 2015-09-25

**Authors:** Amy L. Miller, Matthew C. Leach

**Affiliations:** School of Agriculture, Food and Rural Development, Newcastle University, Newcastle upon Tyne, United Kingdom; University of Maryland, UNITED STATES

## Abstract

Medical research has a heavy and continuing demand for rodent models across a range of disciplines. Behavioural assessment of pain in such models is highly time consuming, thus limiting the number of models and analgesics that can be studied. Facial expressions are widely used to assess pain in human infants. Recently the mouse grimace scale (MGS) has been developed and shown to be accurate and reliable, requiring only a short amount of training for the observer. This system therefore has the potential to become a highly useful tool both in pain research and clinical assessment of mouse pain. To date, the MGS has only been used as a research tool, however there is increasing interest in its use in cage-side clinical assessment. It is often wrongly assumed that MGS scores of animals not in pain (i.e. at baseline) are zero. Here, we aimed to assess the variability in baseline MGS scores between cohorts, sexes and strains of mice. Establishing the presence of a consistent baseline MGS score could lead to a valuable clinical pain assessment tool for mice when baseline information from the individual mouse may not be available as a comparator. Results demonstrated a significant difference in baseline MGS scores between both sexes (males > females) and strains of mice. The method used to score the facial action units (Live vs. retrospectively from still images) demonstrated significant differences in scores with live scores being significantly lower than retrospective scoring from images. The level of variation shown demonstrates the need for further research to be undertaken with regard to establishing baseline MGS scores for specific strains and sexes of mice, taking into account the method of scoring, prior to considering clinical implementation of this method in pain assessment.

## Introduction

Mice are the most commonly used animals in regulated research, with 3.06 million being used in Great Britain during 2012 [1]. This is an increase in numbers from previous years and is largely linked with an increase in breeding of genetically modified (GM) mice. Many of these mice will at some point undergo a potentially painful procedure. Such procedures can range from routine husbandry practices e.g. ear notching [2] through to invasive surgical procedures [3] or can be a potential consequence of their genetic modification [4].

Pain research has a high and continuing demand for rodent models. Although, behavioural-based assessments in such models may offer an effective means of assessing pain, in particular in mice, they can be highly time consuming to develop [3,5] thus limiting the number of models and analgesics that have been studied. Other research techniques that have been used in animal pain research include conditioned place preference [6], self administration [7] and MRI studies [8], however are also time consuming to develop and implement thus again limiting the number of strains and models and subsequently analgesics that can be assessed for effectiveness. In terms of clinical assessment, cage side scoring of behaviour is an effective means of pain assessment in rodents [5,9] but requires trained staff that are familiar with the procedure carried out as well as the species. Well-documented strain variation in the responses of mice to painful stimuli [10] adds an additional layer of complexity to the problem of both pain research and clinical assessment of pain in these animals.

Validated facial expressions are widely used to assess pain in human infants. More recently, the mouse grimace scale (MGS) has been developed [11] and used in the assessment of pain and efficacy of analgesics, in mice following various standard nociceptive and surgical procedures [12,13,14] in research scenarios, i.e. where individual baseline data are available. The MGS consists of 5 facial action units (FAUs) that are independently scored. These are; orbital tightening, cheek bulge, nose bulge, ear position and whisker position. Each is scored on a 3-point scale, and then the sum of all 5 FAUs can be analysed. In these studies, the MGS has been shown to be a highly accurate, repeatable and reliable means of assessing pain requiring only a short period of training for the observer [11,13].

There is increasing interest in the use of the MGS in clinical assessment of pain in mice. However, to date the majority of research studies using the MGS have employed a within-subjects design (i.e. within mouse) where a baseline MGS value (prior to painful stimulus) is available as a comparator. This enables the MGS score observed following a painful stimulus to be compared with a baseline value within each individual and so controls for variation between individuals. Although, this offers an effective means of experimentally validating the MGS for assessing pain, it can inadvertently hide any underlying variation in MGS scores between individuals. Underlying variation, within or between strains, sexes or cohorts of mice may ultimately dictate the value of the MGS for assessing pain in many clinical scenarios, where baseline (prior to pain) MGS values are unlikely to be available. Therefore, we need to establish whether there is a consistent baseline or ‘normal’ level of the MGS scores exhibited by non-painful mice and what level of variation is seen in this baseline score both within (between individuals and sexes) and between common strains of laboratory mice. This is particularly critical for the MGS as to date only a limited number of strains have been assessed using it and baseline MGS scores in these studies are shown to be consistently greater than zero. Only if we can demonstrate a consistent baseline score, which could be applied across scenarios, could this technique be used with confidence as a clinical pain score.

So far, the studies that have used the MGS for pain assessment in research settings, have used images taken (either from video or still photographs) that are assessed at a later time date (i.e. not direct observation). While this is essential in terms of developing and validating this method as a research tool, it does not accurately reflect the potentially changing nature of the face of the mouse in real time or the method that would be applied in clinical scenarios (i.e. direct observation). Therefore, prior to using this method in a clinical scenario, live scoring needs to be deemed consistent and accurate in the same manner as the rodent cage side behaviour scoring documented by Roughan and Flecknell [5,9].

The primary objective of this study was to establish if baseline MGS scores are consistent between cohorts and sexes of mice both within and between strains. Additionally, we aimed to determine if live scoring of facial expressions using the MGS produced the same results as those scored retrospectively from still images.

## Methods

### Animals

Four strains of mice (C57BL/6, C3H/He, CD-1 and BALB/c) aged 7–13 weeks, bred in house or obtained from Charles River UK were used in this study (see Table 1 for full details). Mice were housed in groups of 3–6 with other members of their designated cohort in individually ventilated cages (IVCs) (Arrowmight, Hereford, UK) with sawdust bedding (DBM Ltd, Edinburgh, UK) and nesting material (Sizzle nest, Datesand, UK). Mice were provided with a chew block and cardboard tube (Datesand, UK) for enrichment and a 7-day acclimatisation period prior to the start of the study. The animal room was maintained at 21°C ± 1°C, 48% humidity and on a 12/12 hour light dark cycle (lights on at 07:00). Food (CRM(P), SDS Ltd., Essex UK) and tap water were provided *ad libitum*.

**Table 1:** Details of mice used in this study.

Strain	Cohort	Age (weeks)	Sex	Number of mice	Breeding area
C57BL/6	1	9	Female	7	CBC
	2	8	Female	8	CR
	3	12	Female	7	CBC
	4	8	Female	10	CBC
	5	7	Male	7	CBC
	6	5	Male	10	CR
	7	9	Male	10	CBC
	8	8	Male	10	CR
C3H/He	1	9	Female	10	CR
	2	11	Male	5	CBC
CD-1	1	7	Male	10	CR
	2	8	Female	10	CR
BALB/c	1	8	Female	10	CR

CBC: Comparative Biology Centre, Newcastle University, UK; CR: Charles River UK, Kent

All mice photographed for this study were part of other unrelated research projects so no animals were used solely for this project. No licensed procedures were carried out to collect this data, as it was purely observational. This study was carried out with the approval of Newcastle University Animal Welfare and Ethics Board.

### Sample size

A sample size calculation was carried out using G*Power (V.3.1.) using data from Leach et al [13] and assumed power of 80%. Calculations indicated a minimum sample size n = 6.

### Image collection

Mice were transferred to a quite room for photographing. Mice were placed individually in custom made photography cubes (80 x 80 x 80 mm) which consisted of two clear acrylic sides and two matt, either black or white, acrylic sides which were in contrast to the colour of the individual mouse’s fur. Photographs of the face of the mice were taken across a 10-minute period using a high definition camera (Casio EX-ZR100, Casio Computer Co. Ltd., Japan). Mice were photographed on every occasion they directly faced the camera, apart from when grooming in accordance with the method set out by Langford et al [11].

This process was repeated at three time points for each mouse which represented the most common times of day that mice are checked and assessed by animal care staff; at 9am (AM) representing first thing in the morning, at 12:30pm (Noon) representing lunchtime checks and at 4pm (PM) representing the last checks of the working day. Mice in each cohort were randomly allocated to one of three sequences based upon the time of day (see Table 2). This was done to determine if any change in MGS score for a given cohort was related to the time of day or habituation to the photography boxes.

**Table 2:** Example of order of photography for a cohort of 10 mice. Each cohort is divided into 3 groups (G1, G2 and G3), with the mice being randomly allocated to these groups. Number in brackets refers to the number of individuals in that group.

Session	1	2	3	4	5
Time Point	AM	NOON	PM	AM	NOON
Mice photographed	G1 (3)	G1 (3)	G1 (3)	G1 (0)	G1 (0)
	G2 (0)	G2 (3)	G2 (3)	G2 (3)	G2 (0)
	G3 (0)	G3 (0)	G3 (4)	G3 (4)	G3 (4)

### Image Selection and processing

For each mouse at each time point, 3 photographs were randomly selected from all the clear photographs. Each selected photograph was cropped and only the head of the mouse remained to prevent any bias in scoring of the images due to posture. The selected cropped photographs of each mouse were added to pre-designed excel MGS scoring files in a random order, and were assessed by 3 MGS-trained observers, blinded to all details of the project. For each picture, 5 facial action units (FAUs), orbit tightening, cheek bulge, nose bulge, ear position and whisker position were scored based on the MGS method developed by Langford et al. [11]. Each facial unit is scored on a 3-point scale separately (0 = not present, 1 = moderate, 2 = severe), and the sum of all 5 FAUs were analysed.

### Live Scoring

In addition to photographing the mice, on three separate occasions during the 10-minute recording period an independent female scorer provided a MGS score for each mouse. To generate the score, the observer looked at the mouse for 5 seconds and then awarded a score of 0, 1 or 2 for each Facial Action Unit (FAU) as per the MGS score manual [11]. If the mouse was grooming, the score was recoded following cessation of this behaviour. The mean composite score (i.e. sum) of the five FAUs was used in the further analysis.

### Statistical analysis

Data were analysed using SPSS software (version 21 SPSS Inc, Chicago, IL, USA). The data were analysed non-parametrically. Kruskall-Wallis tests were used to compare multiple cohorts or strains. A Freidman’s test was used to compare groups over time. Mann-Whitney U tests were used to compare the two sexes. Results were considered statistically significant when p < 0.05. An adjusted Bonferroni correction for multiple comparisons was applied where appropriate.

## Results

### Live Scoring

#### Comparison of cohorts

There was no significant difference in MGS score between the four cohorts of male or female C57BL/6 mice at any time of day.

#### Time of day and order effect

There was no significant difference in MGS score between the three time points for C57BL/6, CD-1 or C3H/He mice. BALB/c mice showed a greater MGS score at Noon compared to the AM time point (p<0.05).

None of the mouse strains demonstrated an order effect in the photography boxes with MGS scores remaining constant between the 1^st^, 2^nd^ and 3^rd^ occasions in the box.

#### Comparison of males and females

There was no significant difference in MGS score between the male and female C57BL/6 mice. Male C3H/He mice had a significantly greater MGS score than female C3H/He mice at all three time points (AM: p<0.01, Noon: p<0.001 and PM: p<0.05). Male CD-1 mice had a significantly greater MGS score the female CD-1 mice at the PM time point (p<0.05).

#### Comparison of the strains

**Fig 1:**
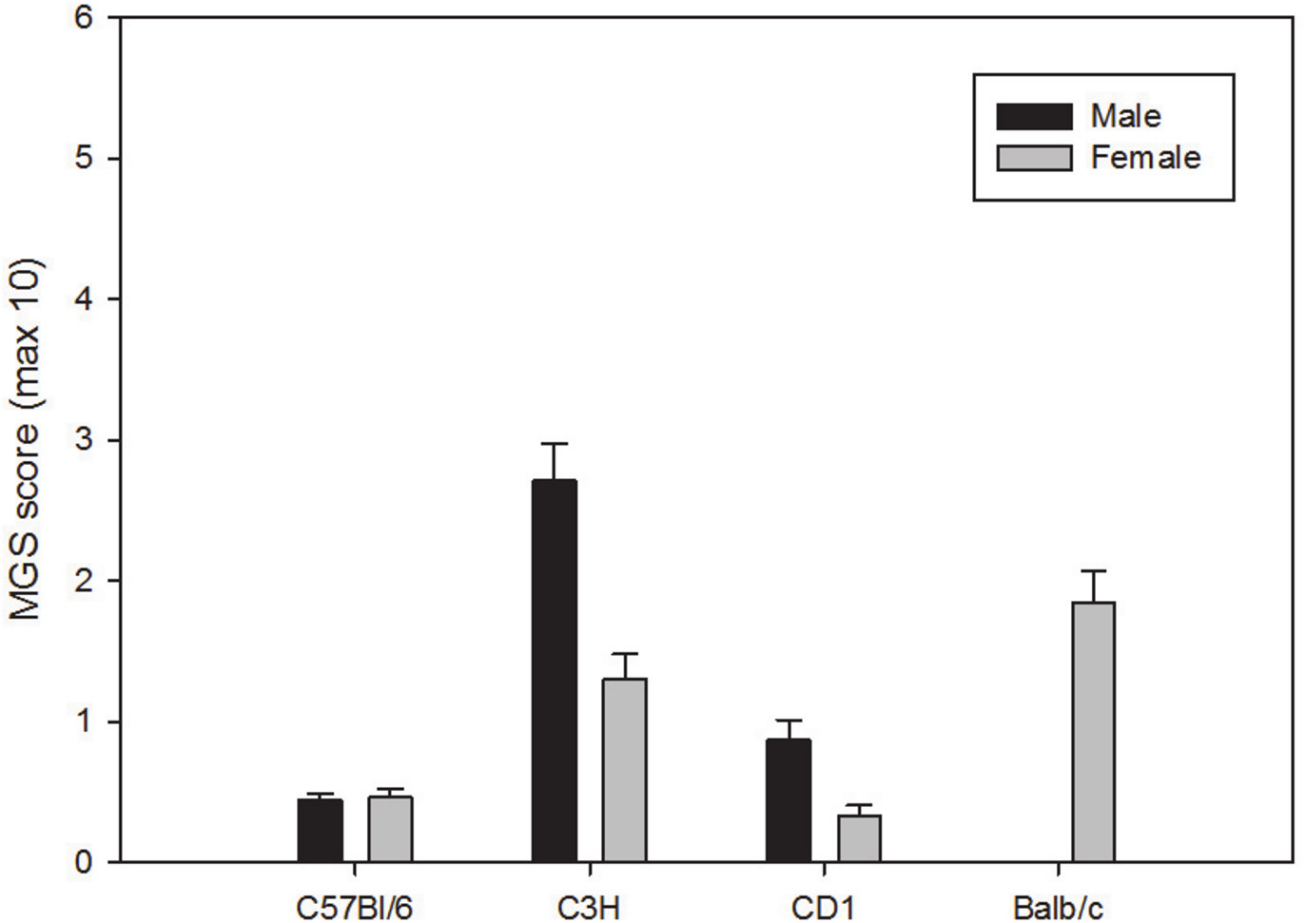
Baseline MGS scores (± SEM) for male and female mice when scored live. Maximum score obtainable 10 (i.e. maximum score of 2 per action unit).

A significant difference was found between MGS scores of the three strains of male mice (C57BL/6 < CD-1 < C3H/He). Significant differences were also found between MGS scores in the four strains of female mice (CD-1 < C57BL/6 < C3H/He < BALB/c) (Fig 1). Tables 3 and 4 shows p values for the significant differences found for males and females respectively.

**Table 3:** Comparison of three strains of male mice, MGS scores. Values shown are p values.

Comparison	P value
C57BL/6 vs C3H/He	<0.001
C57BL/6 vs CD-1	<0.01
C3H/He vs CD-1	<0.001

**Table 4:** Comparison of three strains of female mice, MGS scores. Values shown are p values. NSD represents no significant difference between the strains.

Comparison	P value
C57BL/6 vs C3H/He	<0.001
C57BL/6 vs CD-1	NSD
C57Cl/6 vs BALB/c	<0.001
C3H/He vs CD-1	<0.001
C3H/He vs BALB/c	NSD
CD-1 vs BALB/c	<0.001

### Scoring still images from photographs

#### Comparison of cohorts

There was no significant difference in MGS scores between the four cohorts of male C57BL/6 mice at any time of day. Significant differences were found between the four cohorts of female C57BL/6 mice at both Noon and PM time points. Cohort number 3 had a significantly greater MGS score than cohorts 1, 2 and 4 (p<0.01, p<0.01 and p<0.001 respectively, PM p<0.01, p<0.001 and p<0.001 respectively).

#### Time of day and order effect

There was no significant difference in MGS scores between the three time points for CD-1, C3H/He or BALB/c mice. C57BL/6 mice showed a greater MGS scores at both Noon and PM time points compared to the AM time point (p<0.01 for both comparisons). There was no significant difference, in any strain, between the MGS scores obtained on either the 1^st^, 2^nd^ or 3^rd^ occasion the mice were placed into the photography boxes.

#### Comparison of males and females

There was no significant difference in MGS scores between the male and female C3H/He and CD-1 mice. Male C57BL/6 mice had a significantly lower MGS score than female C57BL/6 mice at all three time points (p<0.05 for all comparisons).

#### Comparison of the strains

There was a significant difference between MGS scores of the three strains of male mice, with C57BL/6 mice having a significantly lower score than both C3H/He and CD-1 mice (p<0.05 for both comparisons). There was no significant difference between the MGS score in the four strains of female mice when assessed using photographs.

#### Comparison of live and photograph scoring

**Fig 2:**
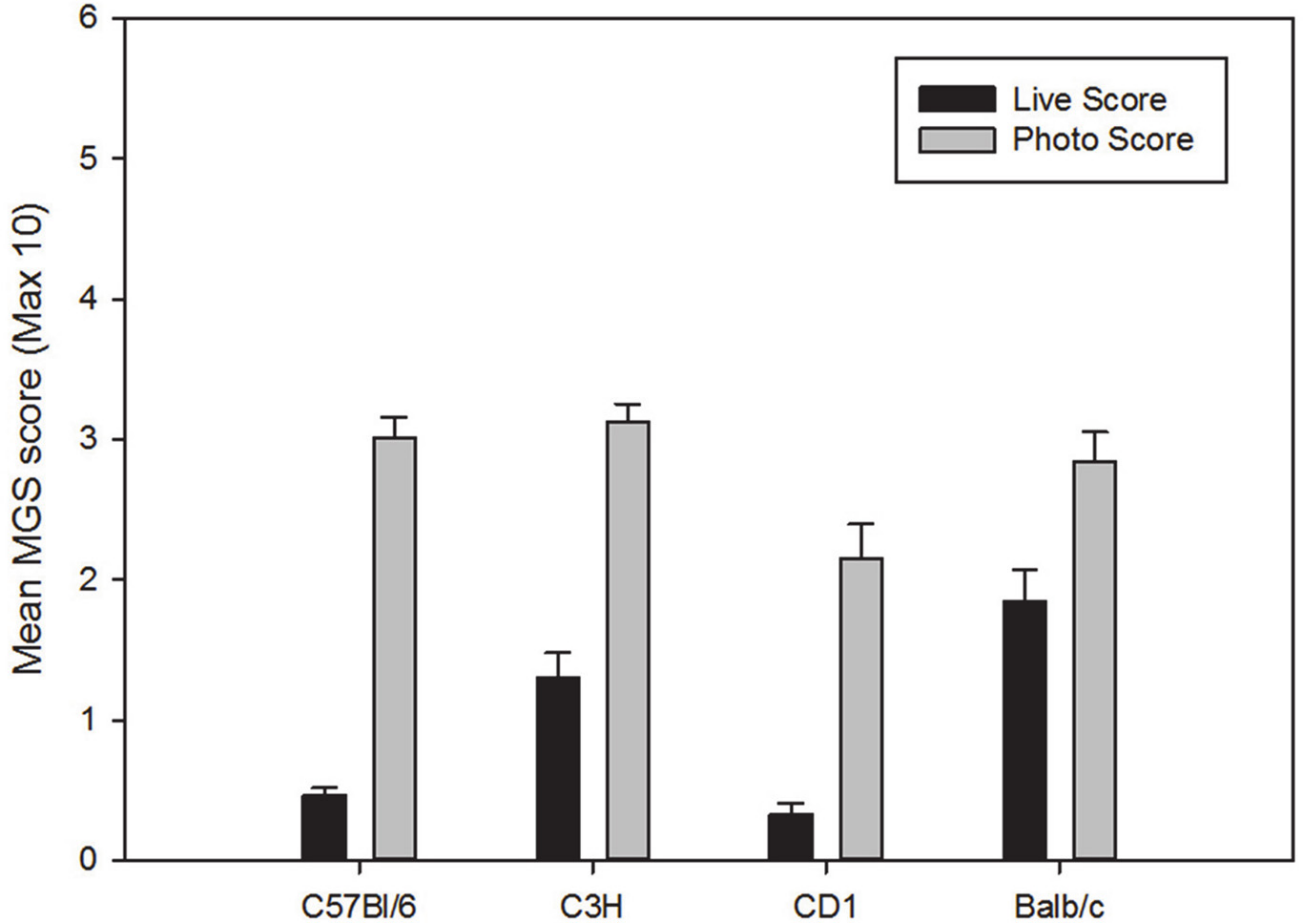
Baseline MGS scores (± SEM) in female mice obtained through live scoring or retrospectively scored from still images. Maximum obtainable score is 10 (i.e. maximum score of 2 per action unit).

**Fig 3:**
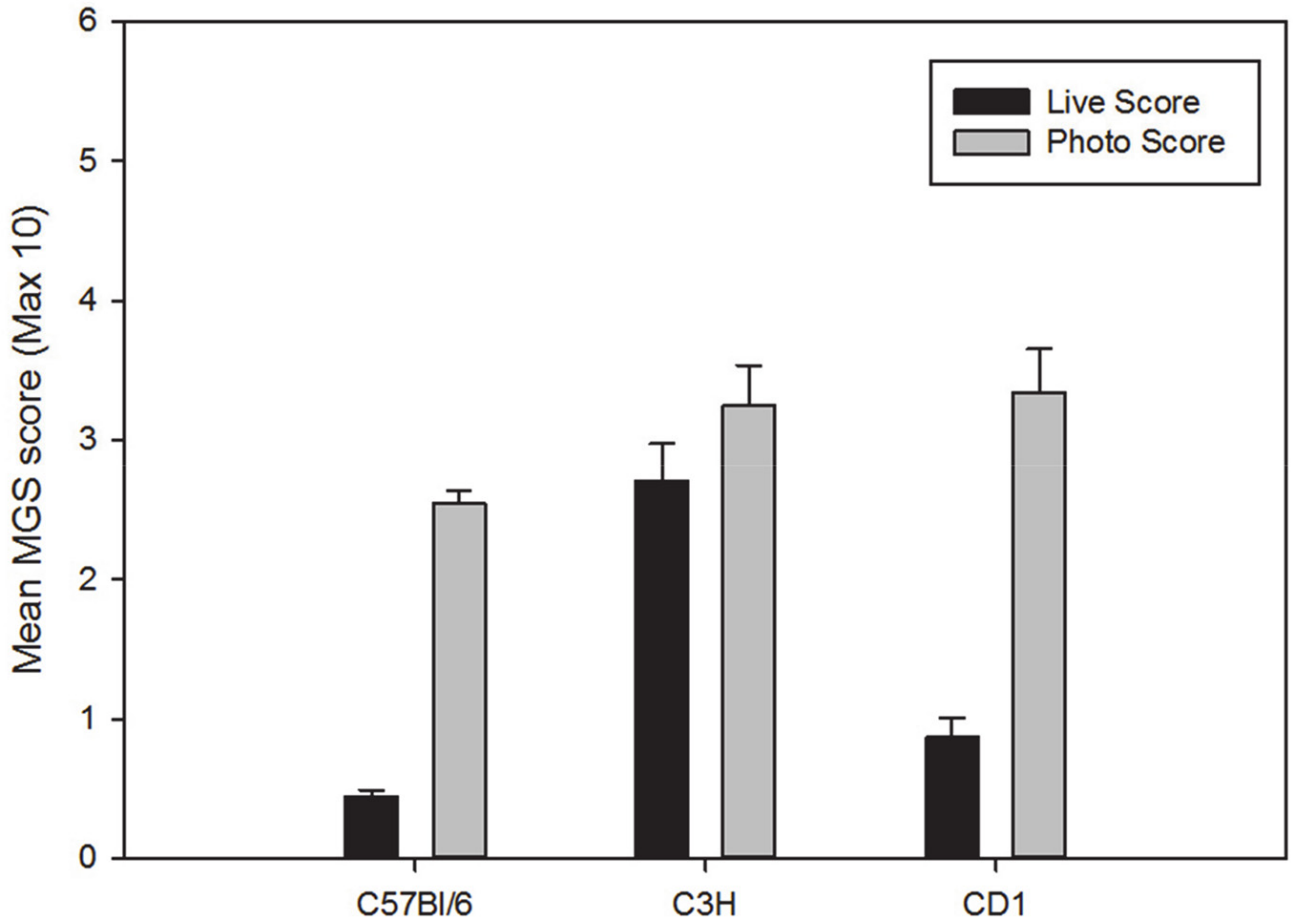
Baseline MGS scores (± SEM) in male mice obtained through live scoring or retrospectively scored from still images. Maximum obtainable score is 10 (i.e. maximum score of 2 per action unit).

Scores obtained from photographs were significantly higher than live scores in all four strains of female mice (C57BL/6: p<0.001, C3H/He: p<0.01, CD-1: p<0.01 and BALB/c: p<0.01) (Fig 2). Scores obtained from photographs were significantly higher than live scores in the male C57BL/6 mice (p<0.001) and CD-1 mice (p<0.01) (Fig 3). There was no significant difference in MGS scores between the two methods in C3H/He male mice.

## Discussion

Ethical and legal requirements regarding the use of mice in research necessitate pain and distress to be reduced to the absolute minimum (Animals (Scientific Procedures) Act 1986, Directive 2010/63/EU). In doing this, scientific benefits are seen by reducing the variability within groups by controlling the variation resulting from uncontrolled pain thus in turn reducing the number of mice required in each study group (i.e. ‘Reduction’). In order to alleviate pain, we must first be able to assess it. Routinely used cage-side indices of pain in mice (i.e. behaviour) have been shown to vary between different procedures and between strains of mice [15,16]. This is of increasing concern due to the expanding use of different strains of genetically modified mice and developments in the range of potentially painful procedures carried out. Therefore, there is a concern that we do not know whether our existing validated pain assessment criteria for mice are still effective in these specific cases.

The MGS is a rapid assessment tool that has shown promise in pain assessment in a research setting [11,13]. There is increasing interest in using this method of pain assessment at the cage-side, in a clinical setting when mice would be scored live, rather than retrospectively from still images and baseline scores for an individual mouse may be unavailable. This study aimed to determine if strain, sex and scoring method influenced the MGS scores in non-painful mice to further determine it's suitability as a pain assessment methods in clinical scenarios.

Limited differences in MGS score were found between different cohorts of the same strain of mice, between three time points (AM, NOON & PM) assessed during the light phase. Additionally no impact of repeated exposure to the photography box was found on MGS score. These are promising findings that demonstrate a level of consistency in the MGS within individual mice and between common mouse strains. This would allow repeated MGS scores, for example, longitudinal monitoring following a potentially painful procedure to be compared without the need for time of day or habituation to the box to be accounted for. This is crucial when developing a pain assessment scale that could be implemented for use in either a clinical or research scenario.

Langford et al [11] noted no significant difference in MGS scores between male and female CD-1 mice. When scoring still images of mice, our data supports this finding for both CD-1 mice and also for C3H/He mice. However, male C57BL/6 mice did show a significantly greater MGS score than females when retrospectively scored from still images. These finding were not observed when mice were scored live. C57BL/6 mice were found to demonstrate no significant difference in MGS score between males and female, whereas C3H/He males had a greater score than females. Male CD-1 mice had a significantly greater MGS score than female CD-1 mice, but only at one time point (PM). Data presented here are in agreement with increasing evidence regarding phenotypic variation observed between different mouse strains in a number of factors e.g. nociception, effectiveness of analgesic compounds and behaviour [10,15,17].

These findings highlight the need for scorers to consider the specific sex and strain of mice they are scoring and also with the method that is employed to collect the data (e.g. live vs. still images). These are all factors that must be taken into consideration when using the MGS.

This study is the first documenting live scoring of the MGS. Live scores, in all the strains used in this study, were always significantly lower than those scores allocated retrospectively from a still image. This could be accounted for by the constant activity and changing nature of the face of the mice when scored live. For example, as a photograph was taken on every occasion the mouse faced the front of the box, by chance on some of these occasions, the eyes of the mouse will be closed (i.e. blinking) providing a score of orbital tightening of greater than 0. These images are then available for random selection and some are likely to end up in the final group to be scored, resulting in an MGS score greater than zero. Whereas when scoring the mice live during a 5 second assessment window, the fact that the mouse has blinked is less likely to result in an increased score in orbital tightening. This variation between methods does have implications for using the MGS in cage-side assessment in a clinical scenario. Environmental conditions may also be worth considering when using the MGS, for example, laboratory temperatures are usually cooler than mice prefer [18] and having aversive lighting [19]. Placing mice in an observation cage away from cage mates, nesting material and often in brighter conditions may have an impact on baseline MGS scores, compared to those that may be obtained through cameras mounted within the home cage. This is a potentially important factor, which to date has not been studied. An additional consideration when transferring this technique to a clinical setting, when prior (baseline) scores for an individual may be unavailable, is that baseline scores are not zero and in some cases; i.e. C3H/He males and BALB/c females scores were greater than 2, a score level that has been observed to be associated with post-surgical pain in CD-1 male mice [13]. In human pain management, patients are often asked to score their pain experience using a numerical rating scale, where 0 is no pain at all and 10 is the worst pain imaginable [20]. Research has demonstrated that when a patient’s score changes by 2 points or more, it is thought to be a clinically important change [21]. This demonstrates the importance of having a baseline score for the individual mouse, or if this is unavailable having prior knowledge to refer to regarding generic baseline scores for the specific strain and sex being studied, otherwise false positive errors (i.e. scoring pain when there is none) could be made. Strain variation prevents us from being able to satisfactorily use one strains baseline score to reflect what may happen in another strain at baseline.

## Conclusion

In conclusion, baseline MGS scores are not zero as is often anticipated. Substantial variation exists between different strains and between the sexes in MGS baseline scores. When scoring mice live, MGS scores are lower than when scoring retrospectively from photographs. These are all factors that must be taken into account when using the MGS to assess pain in mice at the cage-side in a clinical setting when baseline MGS values for each individual are often not available. Further study is required prior to considering clinical implementation of this method in pain assessment to fully establish and validate baseline scores for given strains of mice when scoring is carried out cage-side. Additionally, further study should be undertaken to establish if baseline MGS scores for mice are altered by other factors, such as scoring in their home cage environment instead of photography boxes and when dominant & subordinate mice are together at the time of assessment.

## Supporting Information

S1 File Data used in analysis and used to generate figures. (XLSX)
